# AC-Modulated XPS Enables to Externally Control the Electrical Field Distributions on Metal Electrode/Ionic Liquid Devices

**DOI:** 10.1021/acs.jpcb.4c00152

**Published:** 2024-04-20

**Authors:** Ezgi Kutbay, Suleyman Ince, Sefik Suzer

**Affiliations:** Department of Chemistry, Bilkent University, Ankara 06800, Turkey

## Abstract

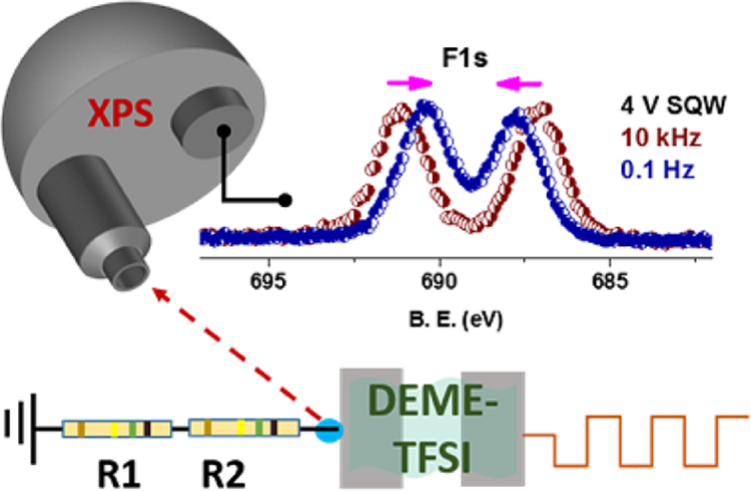

X-Ray Photoelectron Spectroscopy (XPS) has been utilized to extract local electrical potential profiles by recording core-level binding energy shifts upon application of the AC [square-wave (SQW)] bias with different frequencies. An electrochemical system consisting of a coplanar capacitor with a polyethylene membrane (PEM) coated with the Ionic Liquid (IL) *N*,*N*-diethyl-*N*-methyl-*N*-(2-methoxyethyl) ammonium bis(trifluoromethanesulfonyl)imide (DEME-TFSI) as the electrolyte is investigated. Analyses are carried out *in operando*, such that XPS measurements are recorded simultaneously with current measurements. ILs have complex charging/discharging processes, in addition to the formation of Electrical Double Layers (EDL) at the interfaces, and certain properties of these processes can be captured using AC modulation within appropriate time windows of observation. Herein, we select two frequencies, namely, 10 kHz and 0.1 Hz, to separate effects of the fast polarization and slow migratory motions, respectively. Moreover, the local potential developments after adding two equivalent series resistors at three different physical positions of the device have been carefully evaluated from the binding energy shifts in the F 1s peak representing the anion of the IL. This circuit modification allows us to quantify the AC currents passing through the device, as well as the system’s impedance, in addition to revealing the potential variations due the IR drops. The complex AC-modulated local XPS data recorded can also be faithfully reproduced using the unmodulated F 1s spectrum and by convoluting it with electrical circuit output provided by the LT-Spice software. The outcome of these efforts is a more realistic equivalent circuit model, which can be related to chemical/physical makeup of the electrochemical system. An important finding of this methodology emerges as the possibility to induce additional local electrical field developments within the device, the directions of which can be reversed controllably.

## Introduction

Ionic liquids have served as a sustainable alternative to organic solvents for several decades, primarily due to their comparatively low toxicity and nonflammable nature.^1−3^ These remarkable compounds are often referred to as “designer solvents” because they consist of an organic/inorganic cation paired with an anion, offering a platform for tailored functionality across a wide range of applications.^4,5^ Their wide electrochemical window and reasonable electrical conductivity also make them a popular choice in various electrochemical applications, particularly as electrolytes.^6,7^ In addition, their high biological activity has led to their adoption in biosystems, including applications in drug development and delivery.^8^ Ionic liquids possess additional properties that have garnered substantial interest. These properties include low vapor pressure, reduced volatility, and high thermal conductivity,^9,10^ which have prompted growing enthusiasm for their application in conventional areas like energy storage and conversion and their use in batteries, fuel cells, and supercapacitors.^11−17^

The movement of ions between electrified electrodes immersed in liquid electrolytes, such as ionic liquids, is a fundamental process that significantly influences the performance of a wide array of electrochemical systems, spanning various length scales from micro- to macrodimensions. At the core of this phenomenon lies the formation of what is known as the Electrical Double Layer (EDL).^18^ The study of EDL formation with both its static and dynamic characteristics has captivated researchers for nearly two centuries, dating back to the era of Helmholtz.^19−21^ The EDL, whether influencing Faradaic or non-Faradaic processes, emerges as the pivotal factor as ions near charged or polarized surfaces reorganize to counteract the electrode’s charge. This reorganization sets the stage for changes in ion concentration and electrical potential profiles at the interface between the electrode and electrolyte, triggering a complex interplay of kinetic and thermodynamic processes within electrochemical systems.^22^ Recent advancements in EDL formation in ionic liquid systems have revealed multiple time constants and relatively large length scales, up to 10 μm, at solid–liquid interfaces.^23,24^

To investigate the fundamentals of these processes, researchers have used techniques such as electrochemical methods, microscopy, terahertz imaging, and X-ray analysis,^25−36^ often complemented by modeling and simulations.^37,38^ Still, every technique has its own shortcomings when it comes to replicating real-world electrochemical devices. Another tool often utilized for deeper insight is X-Ray Photoelectron Spectroscopy (XPS).^39−44^ Over the course of several years, our research group has harnessed the power of XPS to delve into the intricate details of ionic liquid/metal interfaces. XPS, renowned for its exceptional surface sensitivity, enables us to unravel some of the chemical and electronic properties of the samples and/or devices. This technique offers a probing depth that ranges from 1 to 8 nm, allowing one to determine element- and chemical-specific properties and the relative quantities of them on the sample’s surface.^45^ One emerging and promising application of XPS within our field involves directly extracting the local electrical potential profiles within electrochemical systems. Since most of the electrochemical techniques utilize current (amperometric) measurements, additional information from the potential profiles (voltammetric) complements and possibly offers new observation channel(s).

XPS excels in measuring the binding energy shifts of core-level photoelectrons in a chemically specific manner, particularly under electrical bias, establishing it as a minimally invasive technique. In typical lab-based instruments, the binding energy uncertainty is down to ∼20 meV. Even in the presence of the currents associated with the photoelectron emission process, usually around ∼1 nA, the resulting binding energy shift (due to the IR drop) remains minimal, roughly about 1 meV, a value well below the threshold of experimental error. This effect proves to be inconsequential, particularly when considering a typical ionic liquid film device having an overall intrinsic resistance of approximately 1 MOhm.

We have demonstrated that employing a DC bias during XPS data collection provides us with stable, steady-state information.^46−49^ Furthermore, our research has emphasized the essential role of the AC excitation in examining dynamic evolution of the electrical potentials, as showcased in various of our publications.^50−54^ Recently, we also reported on the use of Scanning Electron Microscopy (SEM) to detect similar changes caused by potential-induced intensity variations.^55^ Notably, these measurements have enabled us to observe the effects of time-resolved polarization and screening of metal electrodes into the liquid electrolyte surfaces over significant distances (centimeters) and extended time periods (hundreds of seconds), offering insightful chemical information.

Recently, we have extended our research to explore characteristics of the diffuse dynamics within a coplanar capacitor setup, utilizing ionic liquids (ILs) as the electrolyte.^56^ This investigation combined XPS and electrochemical measurements while subjecting the system to square-wave (AC) potential excitation across a range of frequencies, from mHz to kHz, all of which were conducted at room temperature. The coplanar capacitor configuration allows us to spatially extend the X-ray beam and probe locally the effects of screening of the applied potential through ionic movement. We aimed to investigate the impact of ionic motion across the electrolyte medium, discerning how it screens the applied potential throughout the entire device. This study was crucial, as ionic motion influences all regions of the system, making it a prevalent factor in our analysis.

Our most recent work along these lines had extended the investigation of the same hybrid electron-ion conducting device after incorporating two equivalent external series resistors within the ultrahigh-vacuum chamber of the instrument. As in most cases, we record the strong F 1s spectrum of the IL film at different local positions on the entire device, covering both the electrode surfaces and insulating membrane that connects the electrodes.^57^ The IL film is about 10–50 μm thick on the electrodes and provides the conduction, while the device is subjected to square-wave-AC modulation with two different frequencies, namely, the high (10 kHz) and low (0.1 Hz) frequencies. In that work, we had determined the AC resistance (impedance) values of the device under the high and low frequencies to be ∼300 and ∼580 kOhm, respectively, i.e., increasing by a factor of 2 under the low frequency. Furthermore, we had surprisingly found that, after inserting the series resistors, the direction of the potential screening was reversed between the two frequencies. In this contribution, we delve deeper into our investigation to better understand this surprising reversal of the direction in the potential screening by shifting the physical position of the series resistors and determining their effects on the recorded AC-modulated XP spectra. As in our previous work, we also employ the LT-Spice software for electrical modeling and to reproduce the local AC-modulated F 1s peaks and compare them with the recorded XPS data, which enabled us to obtain a much more realistic equivalent circuit of the device.

## Experimental Section

**Figure 1:**
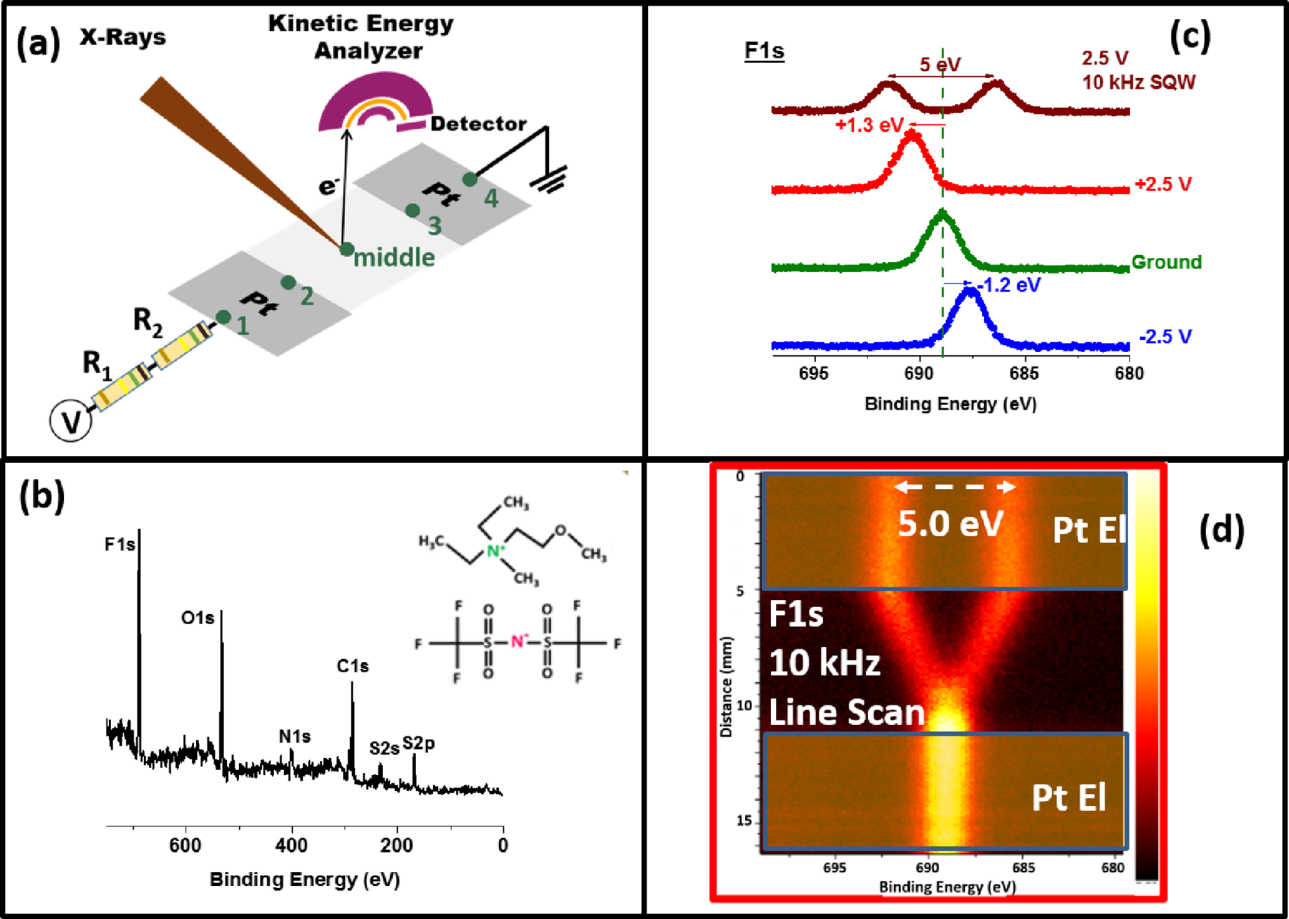
(a) Schematics of the coplanar capacitor device with two series resistors (R1 and R2) for investigating the charging dynamics of the ionic liquid electrolytes using XPS under external bias. The function generator is connected to the source electrode to impose either a DC or AC (square-wave) excitation with fixed or variable frequencies. (b) Survey XP spectrum of the IL and its chemical formula is given as the inset. (c) XP spectra of the F 1s region, recorded at the source electrode under different biasing conditions (0, + 2.5, and −2.5 V DC), as well as under 2.5 V 10 kHz square-wave modulation, without the additional resistors. (d) The same 2.5 V SQW 10 kHz modulated 108 F 1s XP spectra are depicted as a line scan starting from the first Pt electrode (point **1**) and going in the middle all the way to the end of the second Pt-grounded electrode (point **4**). Shaded regions correspond to the metal electrodes.

The coplanar capacitor configuration employed in our previous and in this study consists of two platinum (Pt) electrodes deposited onto a porous polyethylene membrane (PEM), with one serving as the source (working electrode) and the other as the drain (counter electrode). As in our previous investigations, we have chosen the ionic liquid, *N*,*N*-diethyl-*N*-methyl-*N*-(2-methoxyethyl) ammonium bis(trifluoromethanesulfonyl)imide, (DEME-TFSI), since it is the prototypical system for exploring various electrochemical applications.^58−62^ Moreover, the sizes of the anion and cation are comparable. A 5 μL volume of the ionic liquid is applied to the membrane, creating a continuous liquid film as the electrolyte medium. Additional series resistors are inserted into the setup, as illustrated in Figure 1a, and also in two other configurations.

XPS measurements are recorded using a Thermo Fisher K-Alpha XP spectrometer with a 50 eV analyzer pass energy. For time-resolved XPS, a snapshot mode with a 150 eV pass energy is used for faster data acquisition instead of the scanning mode. The XP survey spectrum of the ionic liquid is presented in Figure 1b, and the chemical formula of the ionic liquid is also provided as an inset. Figure 1c displays the XP spectrum of the F 1s region under −2.5, 0, and +2.5 V DC bias conditions as well as under 2.5 V square-wave AC modulation at 10 kHz on a point at the surface of the IL film on top of the source electrode. Upon application of positive and negative DC bias of 2.5 V, the F 1s peak shift only +1.3 and −1.2 eV (i.e., approximately half of the bias), respectively, due to screening of the full voltage by the IL film.^50^ Application of the square-wave (SQW-AC) bias results in twinning of peaks, as both positive and negative cycles are imposed simultaneously, as seen in the same figure. However, under 10 kHz modulation, the ionic motion is frozen; hence, the binding energy difference is recorded as 5.0 eV, reflecting faithfully the full bias (2 × 2.5 = 5.0 V) and the absence of any IR drops.

In Figure 1d, we display 108 F 1s XP spectra recorded, using the snapshot mode, and along the line starting from the position **1** and going down the device all the way to the position **4**. As also indicated by the shaded regions, there are 3 distinct regions on the device: region I corresponds to the surface of the IL film on top of the electrified Pt electrode, region II corresponds to the surface of the IL on and below the insulating polyethylene membrane, and region III corresponds to that of the IL film on the grounded Pt electrode. All regions reveal pertinent information about the ion dynamics. For example, in the entire region I, a uniform and full electrical potential of 5.0 V is revealed by the F 1s signals since the ion motion is frozen. This potential is linearly decreased starting from the beginning of region II and diminishes completely (i.e., the twinned F 1s peaks merge into each other) due to the finite resistance of the IL in this region (IR Drop). As a result, no potential variation is measured throughout region III at 10 kHz. However, all of these changes manifest differently in each region under the low 0.1 Hz modulation since ions start to screen the local potentials in time. The two selected AC frequencies reflect the time windows corresponding to the fast predominantly electronic polarization of the bulk ionic liquid at 10 kHz, and to the slow migratory processes, dominated by ionic motion at 0.1 Hz, as discussed by us,^56,57^ which was also recommended by others, who had reported electrochemical investigation of a similar devices.^61,62^ The idea behind is the possibility of separating contributions of the two processes, i.e., bulk electronic polarization vs electrochemical. Accordingly, through the measurements at 10 kHz (i.e., within 0.05 μs time-window), we capture the potential variations of the initial electric field imposed by the bias, which is constant in region I, and linearly decreasing to zero (V-shaped binding energy difference between the twinned F 1s peaks) in region II, and zero everywhere in region III. The overall Y-shaped spectral feature in the regions II and III points out that a net zero effective screening is measured at 10 kHz on the entire device. On the other hand, measurements at 0.1 Hz (i.e., within a 5 s time-window) reflect the local effective screening of the initial polarization. Similar to what was discussed in detail in our previous paper,^57^ we introduce another electrical parameter, namely, two equivalent series resistors in three different geometries; (i) both before the IL device, (ii) one before and one after, and (iii) both after the device. The magnitude of the resistors is chosen to provide an equivalent IR drop to that of the pristine IL device at 10 kHz. Accordingly, the amplitude of the AC bias is increased to ensure that the electrical field strength is comparable to the one in the pristine IL-devices after introduction of the resistors. Therefore, while 3.0 V SQW bias is used for the pristine device, 4.0 V SQW is employed after the resistors are added to the electrochemical system.

## Results and Discussion

### XPS Measurements

#### IL-Co-Planar Device without and with Resistors in Front

**Figure 2:**
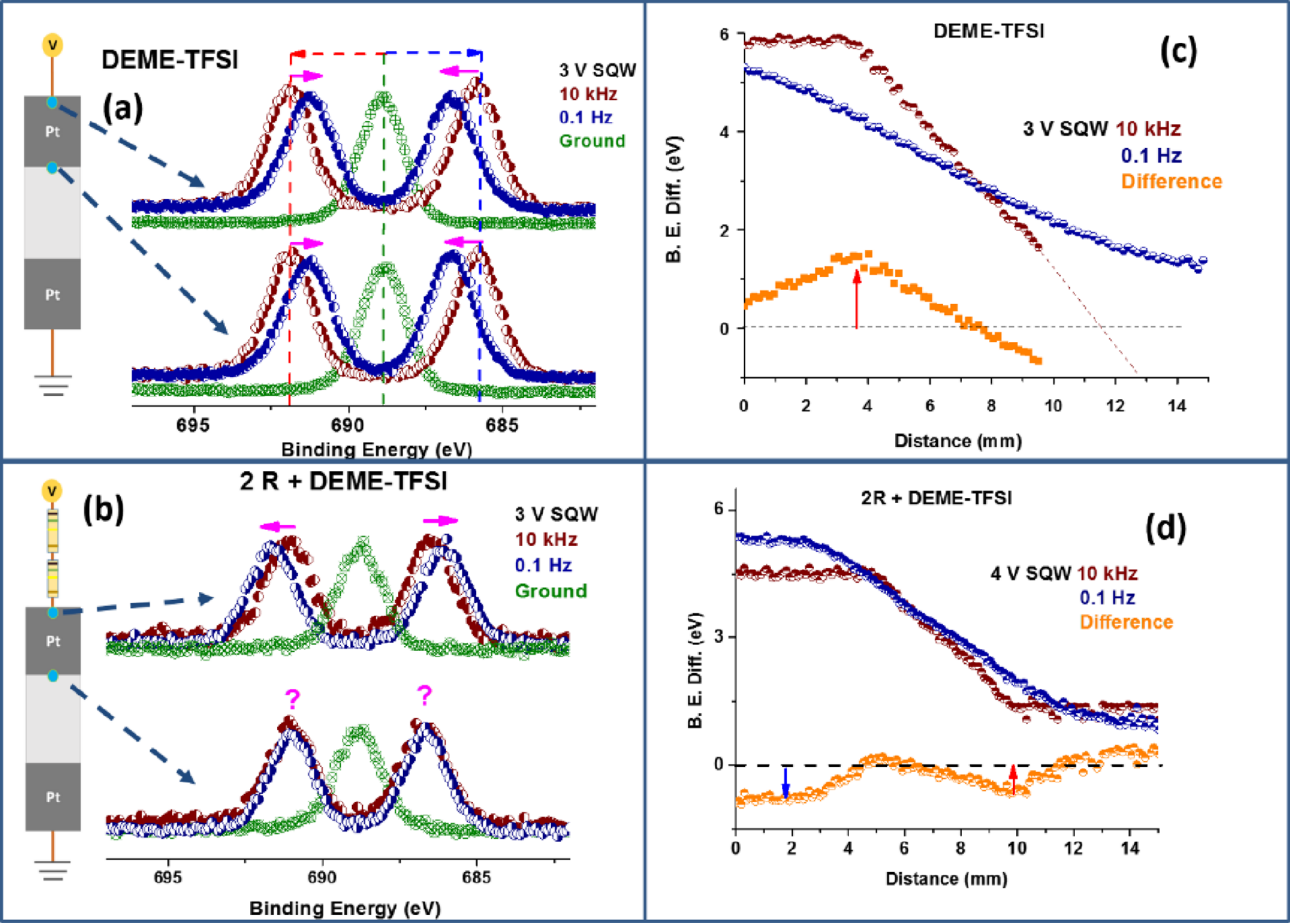
XP spectra of the F 1s region, recorded when grounded (green) and under 2.5 V SQW at 10 kHz (wine) and at 0.1 Hz (royal blue) at the beginning and at the end of the electrified Pt electrode; (a) before and (b) after addition of the two resistors in front. Measured local electrical potential variations along the entire device, derived from the binding energy difference between the twinned peaks, are plotted at the two frequencies, as well as the differences between them; (c) IL-only device, and (d) after introduction of the resistors.

Figure 2 displays a number of representative measurements of the pristine IL device after outgassing and heating in a vacuum. In Figure 2a, we show the F 1s region’s spectra recorded under 3.0 V SQW excitation at the two frequencies and at the beginning of the biased electrode [position **1** of Figure 1a] and at the end (position **2**). Figure 2b gives the corresponding F 1s spectra after two resistors are introduced before the IL device and under 4.0 V SQW excitations. As also indicated in the figures, at the beginning of the electrified electrode (point **1**) the direction of the shifts has been reversed after introducing the series resistors.

This important observation reveals that the direction of the AC electric field can be reversed by the addition of external series resistors. A naïve interpretation can be offered as follows; while the screening of the local ions near the very beginning and near the end of the biased electrode are effectively decreasing the potential imposed by screening it, when an extra 5 s is allowed (0.1 Hz) for them, introduction of the serial resistors causes them to act in the opposite directions, such that the ions now help to increase the imposed bias.

In Figure 2c,d, we display the local electrical potential variations at the two frequencies, extracted from the difference in F 1s binding energies of the twinned F 1s peaks, recorded along the entire device at the two frequencies, as well as the difference between them for the devices before and after introducing the series resistors, respectively. A closer examination of Figure 2c,d reveals that the largest potential screening is observed at the metal/insulating membrane interface, which diminishes locally after introduction of the resistors. Demonstrating the possibility of controlling externally the local electrical field(s).

#### IL-Co-Planar Device in between the Resistors and in Front of Them

**Figure 3:**
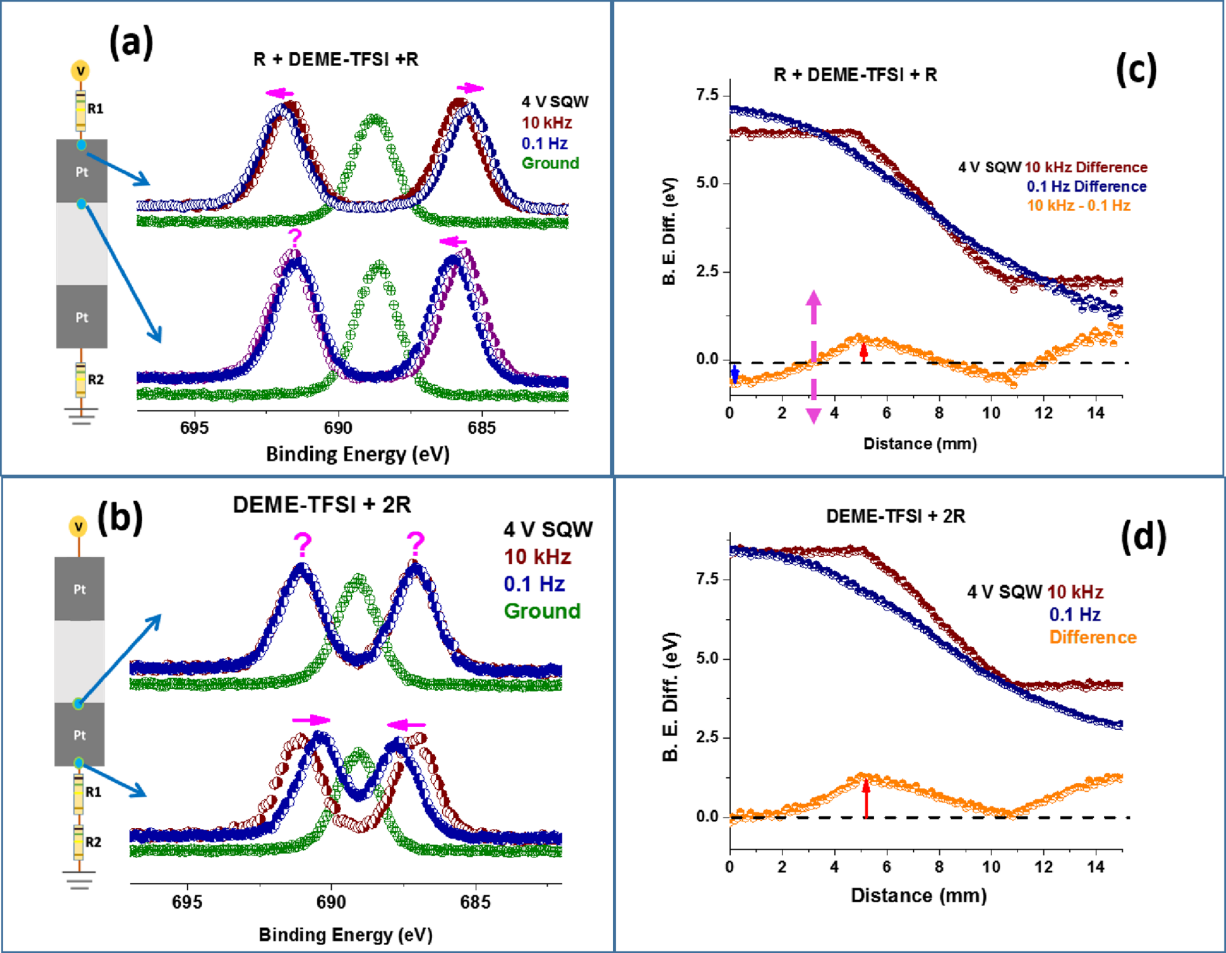
F 1s region, recorded when grounded (green) and under 4.0 V SQW at 10 kHz (wine) and at 0.1 Hz (royal blue) at the beginning and at the end of the electrified Pt electrode, when two resistors are added; (a) one in-front and one in-back, and (b) both in-back. Measured local electrical potential variations along the entire device, derived from the binding energy difference between the twinned peaks, are plotted at the two frequencies, as well as the differences between them, when two resistors are added; (c) one in-front and one in-back, and (d) both in-back.

We have continued our investigation by placing the device between the two resistors and also in front of them. Representative spectra and the data extracted from them are displayed in Figure 3. In Figure 3a, we show the F 1s region’s spectra recorded under 4 V SQW excitation at the two frequencies, and after placing one resistor before the biased electrode and the other one after the grounded one. Figure 3b gives the corresponding F 1s spectra when two resistors are introduced after the IL device. This time representative spectra are displayed in the grounded electrode region since they are more informative. The corresponding local electrical potential variations are plotted at the two frequencies in Figure 3c,d, respectively.

As also indicated in Figure 3c, at the beginning of the electrified electrode (point **1**), the direction of the shifts has been reversed after sandwiching the IL device between the series resistors and only in the negative cycle of the SQW excitation. In addition, for the same device, there is a point in the middle of the electrified electrode that the electric field difference is diminished, like a neutrality point where the ionic screening at the low frequency is canceled by the electrical field imposed by the higher frequency. Similar electrical field reversals/variations are transferred to the formerly grounded electrode when the resistors are placed at the end of the device, which are depicted in Figure 3d. The corresponding experimental data are collected in Table1.

**Table 1:** Measured F 1s Peak Positions at Different Locations and under Various Excitations

		**binding energy (eV)**				
parameter	location	3.0 V 10 kHz	**Diff.**	3.0 V 0.1 Hz	**Diff.**	**Grnd**
**IL device**	Pos. **1**	691.8 685.8	6.0	691.2 686.6	4.6	688.8
	Pos. **2**	691.8 685.8	6.0	691.3 686.6	4.7	688.8
	Pos. **3**	688.8 688.8	0.0	689.6 688.1	1.5	688.8
	Pos. **4**	688.8 688.8	0.0	688.9 688.9	0.0	688.8
		4.0 V 10 kHz	**Diff.**	4.0 V 0.1 Hz	**Diff.**	**Grnd**
**R_1_ + R_2_ + IL device**	Pos. **1**	686.5 691.1	4.6	686.1 691.6	5.5	688.8
**simulation**		687.0 691.8	4.8	686.2 692.5	6.3	
	Pos. **2**	686.7 691.0	4.3	686.7 691.0	4.3	688.9
**simulation**	Pos. **2**	687.0 691.8	4.8	687.1 691.6	4.5	
**R**_**1**_**+ IL device + R**_**2**_	Pos. **1**	685.8 691.7	5.9	685.5 692.0	6.5	688.7
**simulation**	Pos. **1**	686.1 692.5	6.4	685.8 692.9	7.1	
	Pos. **2**	685.7 691.7	6.0	686.0 691.4	5.4	688.7
**simulation**	Pos. **2**	686.1 692.5	6.4	686.7 692.0	5.3	
**IL device + R**_**1**_**+ R**_**2**_	Pos. **3**	687.1 691.0	3.9	687.1 691.1	4.0	689.1
**simulation**	Pos. **3**	687.7 690.9	3.2	687.6 691.1	3.5	
	Pos. **4**	687.1 691.1	4.0	687.7 690.4	2.7	689.1
**simulation**	Pos. **4**	687.7 690.9	3.2	688.8 690.2	1.4	

All of these observations reveal that the direction of the AC electric field can be controlled at will via a very unique experimental tool for extracting certain electrical properties of the systems analyzed.

### Modeling and Equivalent Circuits

**Figure 4:**
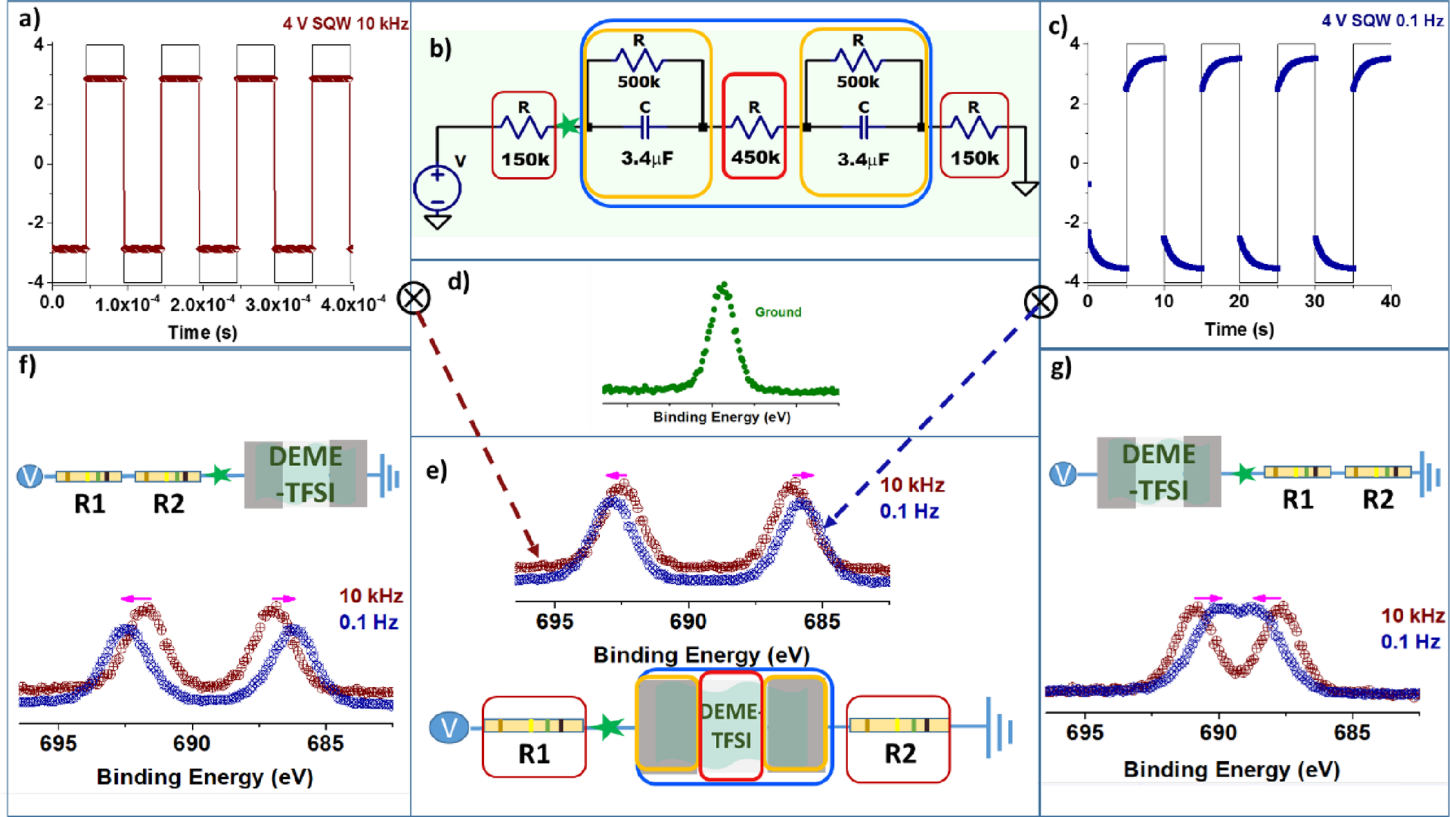
Simulation of the F 1s XP spectra via LT-Spice software for the three different geometries used in this work. (b) Schematics of the equivalent circuit used. The voltage output of the LT-Spice software is shown in panels a and c for the 4 V SQW under 10 kHz and 0.1 Hz, respectively. The voltage outputs generated are then convoluted with the F 1s spectrum recorded without any bias, which is shown in panel d. The resulting synthetic spectra at the 2 frequencies and for the 3 different geometries used in this work are depicted in panels e–g, respectively.

As we have often reported in our previous work, we utilize the commonly consulted LT-Spice simulation program to combine its output to reproduce synthetic XP spectra and compare them with the those obtained from measurements.^57^ Essentially, we start with an approximate equivalent circuit constructed using the measured current values and other known electrical parameters about the device, and the voltage output provided by the program, at different local positions along the device and at the designated frequencies are convoluted with the IL’s F 1s region’s XP spectrum recorded under no bias, to mimic the spectra obtained under AC modulations as schematically shown in Figure 4. This procedure is iterated by varying the R and C values until we have reasonable consistency between the synthetic spectra produced by the program and the XPS spectra recorded.

To implement it to our new geometries, we use the results of the electrochemical impedance measurements from our previous work, which were determined as the overall capacitance and resistance values as 1.7 μF and 450 kOhm, respectively.^57^ Moreover, an additional parallel resistor of ∼1 MOhm value was then needed to match the XPS data since we had also learned that the capacitance of the device is predominantly determined by the two metal/IL interfaces, meaning that capacitance corresponding to the region II between the electrodes is negligible, we split the RC part of the circuit into two parts. In doing so, we also divided the resistances into two and doubled the capacitances to keep the overall time constant the same, and the finalized circuit for the device having the series resistors is shown in Figure 4b. The resulting synthetic spectra at the corresponding local positions for the three device configurations are reproduced in Figure 4e–g.

In Table 1, we have also added the positions of the twinned peaks of the synthetic spectra obtained from the simulations and also the shifts at the designated points in Figure 4. In most cases, they are in harmony with the XPS data, with few exceptional cases like at the point 4 of the device, which has both resistors in the back. Considering the simplicity of our simulation route and the complexity of the electrified ionic liquids’ dynamics, we conclude that the approach is satisfactory, but definitely needs further improvements, such as more experimental precision in fabrication of the devices, and/or more care in mitigation of the unwanted impurities.

## Conclusions

Entangling the chemical and physical parameters affecting the complex dynamics of the electrical potential variations in and around ionic liquid/metal interfaces is a colossal task and requires orchestrating the use of a multitude of modern electrical, spectroscopic, and microscopic experimental and theoretical tools. In this work, we introduce a simple and novel variant of the XP-spectroscopic technique to effectively control and capture the electrical field direction changes by introducing solid-state circuit elements, which is relatively simple and noninvasive. Herein, we utilized this tool to record AC-modulated XP spectra at two frequencies and compared them with the LT-Spice software output to construct a more realistic equivalent circuit model. Further utilization of the technique by us and others is expected to impact significantly on better understanding of the dynamics of electrochemical systems at the atomic and molecular level and help develop next-generation energy storage and harvesting and sensing devices.
